# Improved thermostability of an acidic xylanase from *Aspergillus sulphureus* by combined disulphide bridge introduction and proline residue substitution

**DOI:** 10.1038/s41598-017-01758-5

**Published:** 2017-05-08

**Authors:** Wenhan Yang, Yongzhi Yang, Lingdi Zhang, Hang Xu, Xiaojing Guo, Xu Yang, Bing Dong, Yunhe Cao

**Affiliations:** 1 State Key Laboratory of Animal Nutrition, China Agricultural University, No.2 Yuanmingyuan West Road, Beijing, 100193 China; 2 School of Medicine Biochemistry and Molecular Genetics, University of Colorado Denver, Aurora, CO 80045 USA; 3 Institute of Biophysics, Chinese Academy of Sciences, Beijing, 100101 China

## Abstract

As a feed additive, xylanase has been widely applied in the feed of monogastric animals, which contains multiple plant polysaccharides. However, during feed manufacture, the high pelleting temperatures challenge wild-type xylanases. The aim of this study was to improve the thermostability of *Aspergillus sulphureus* acidic xylanase. According to the predicted protein structure, a series of disulphide bridges and proline substitutions were created in the xylanase by PCR, and the mutants were expressed in *Pichia pastoris*. Enzyme properties were evaluated following chromatographic purification. All the recombinant enzymes showed optima at pH 3.0 and 50 °C or 55 °C and better resistance to some chemicals except for CuSO_4_. The specific activity of the xylanase was decreased by introduction of the mutations. Compared to the wild-type enzyme, a combined mutant, T53C-T142C/T46P, with a disulphide bond at 53–142 and a proline substitution at 46, showed a 22-fold increase of half-life at 60 °C. In a 10-L fermentor, the maximal xylanase activity of T53C-T142C/T46P reached 1,684 U/mL. It was suggested that the T53C-T142C/T46P mutant xylanase had excellent thermostability characteristics and could be a prospective additive in feed manufacture.

## Introduction

Xylan, present in plant cell walls and middle lamella, is the most abundant natural cell wall polysaccharide next to cellulose^1^. Xylanase (EC 3.2.1.8) degrades xylan by randomly hydrolysing the β-1,4-glycosidic bonds to produce xylo-oligosaccharides of different lengths^2^. Thus, xylanase has been widely applied in the industries of animal feeding, food baking and pulp bleaching, among others^3–5^. Due to the technical requirements during processes such as feed pelleting and food baking, people need higher-thermal-capacity xylanase. Scientists therefore have developed multiple means of improving the thermostability of xylanase.

An earlier means was to clone hyper-thermophilic xylanase from a variety of micro-organism sources, such as *Thermotoga maritima* MSB8^6^, *Caldocellum saccharolyticum* ^7^, *Geobacillus sp*. MT-1^8^ and *Thermotoga neapolitana* DSM 4359^9^. Later means were based on rational design and directed evolution methods to improve the properties of xylanases. Satyanarayana^10^ enhanced the thermostability of xylanase (MxylM4) by substituting serine/threonine with arginine residues by site-directed mutagenesis. Wang *et al*.^11^ improved the thermal performance of *Thermomyces lanuginosus* GH11 xylanase by introducing a disulphide bridge, Q1C-Q24C, into the N-terminal region of the enzyme.

In our laboratory, an acidic β-1,4-xylanase gene *xynA* was cloned from *Aspergillus sulphureus* and constitutively expressed in *Pichia pastoris* ^12, 13^. The recombinant enzyme showed high catalytic activity and stability at extreme acidic pH values, which is superior in passing stomach. However, its thermostability still needs to be improved at temperatures over 60 °C. In this study, we created xylanase mutants by introducing a disulphide bridge and proline residue substitutions to improve the enzyme’s thermostability.

## Results

### Construction of xylanase mutants and expression in *P. pastoris*

**Figure 1:**
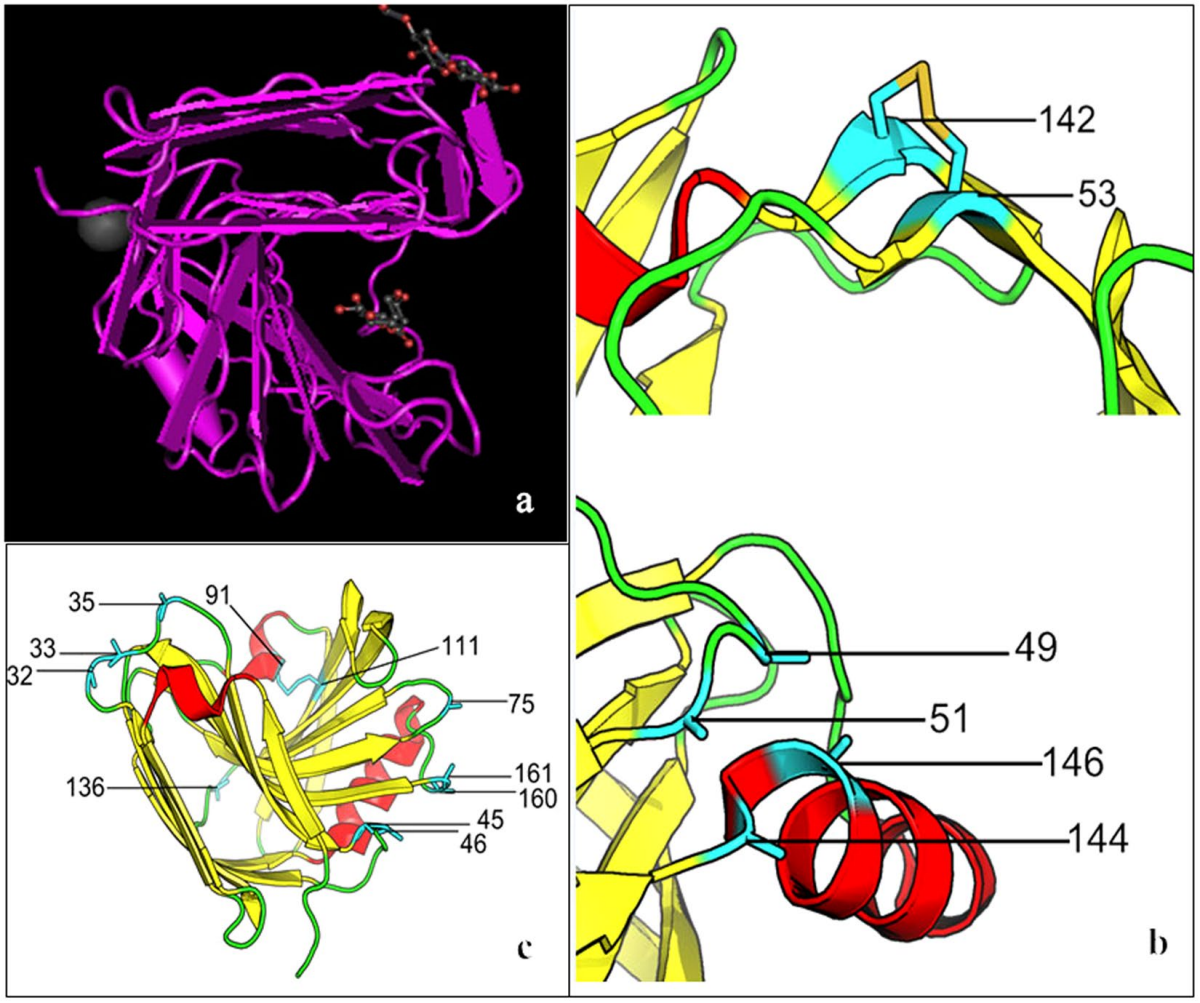
The positions of the designed disulphide bridges and proline substitution sites in *Aspergillus sulphureus* xylanase. (**a**) Model structure of xylanase (PDB 2QZ2_A) for the prediction of mutations. (**b**) The disulphide bridge at residue positions 53 and 142 is shown in blue; the β-sheets are shown in yellow; α-helixes are shown in red and the random coils are shown in green. The residue positions 49, 51, 144 and 146 are shown in blue. (**c**) The positions of introduced proline residues in *A. sulphureus* xylanase. The proline residues at sites 32, 33, 35, 45, 46, 75, 136, 160 and 161 are shown in blue, the β-sheets are shown in yellow, α-helixes are shown in red and the random coils are shown in green. The native disulphide bridge 92–111 of the wild-type xylanase is shown in blue.

In this study, two types of mutations were introduced into the wild-type *A. sulphureus* xylanase to improve its thermostability. One was the introduction of disulphide bridges into the enzyme molecule, based on its homologously modeled structure. First of all, residue pairs with close distances were identified, and mutated *in silico* to cysteine. The proximity and geometry of these cysteine-pairs were individually assessed in Coot^14^, to ensure proper disulphide formation. The resulted 49 candidate pairs were further examined by the following criteria: (1) the paired residues should be separated in the primary sequence, as a local linkage only provides limited stabilization; (2) the paired residues should not be involved in or close to the binding pocket, otherwise the enzymatic activity might be negatively affected. The potential disulphides were screened for their pairing possibility and distance to the active center. Following the above rational, three pairs of potential sites (S49 and A146, P51 and T144, T53 and T142) were selected for substitution with cysteine residues to construct potential disulphide bridges (Fig. 1). The engineered variants, S49C-A146C, P51C-T144C and T53C-T142C, were all obtained.

The other was to substitute proline for certain amino acid residues at random coils in the enzyme structure. The structural model was carefully examined and substitution sites were selected at sharp backbone turns, expecting prolines provide better stabilization to these turns. A total of 9 amino acid residues, including D32, G33, S35, T45, T46, Y75, S136, S160 and D161, were selected for substitution with proline (Fig. 1), namely Mutants D32P, G33P, S35P, T45P, T46P, Y75P, S136P, S160P and D161P, respectively.

Based on the thermostability of the single mutations, we selected three mutants (T53C-T142C, T46P and S136P) that increased xylanase activity the most to construct four combined mutants (three double mutations and one triple mutation). Among these three double mutations, two mutants combined one disulphide bridge pair (T53C-T142C) with a proline substitution (T46P or S136P) to generate Mutants T53C-T142C/T46P and T53C-T142C/S136P, respectively. Mutant T46P/S136P contained two proline residue substitutions at T46 and S136. Mutant T53C-T142C/T46P/S136P carried four single-amino acid mutations at positions 53, 142, 46 and 136 to form a disulphide bridge and two proline substitutions. There was no free sulfhydryl in T53C-T142C/T46P, T53C-T142C/S136P, and T53C-T142C/T46P/S136P mutants (Table 1), which indicated that the disulphide bridge pairs were formed as designed.

**Table 1:** The quantity of the free sulfydryl in the mutants.

Mutant	T53C-T142C/T46P	T53C-T142C/S136	T53C-T142C/T46P/S136P
free sulfydryl quantity	0	0	0

DNA sequencing confirmed that the constructed mutants DNA sequences were the same as its design.

**Figure 2:**
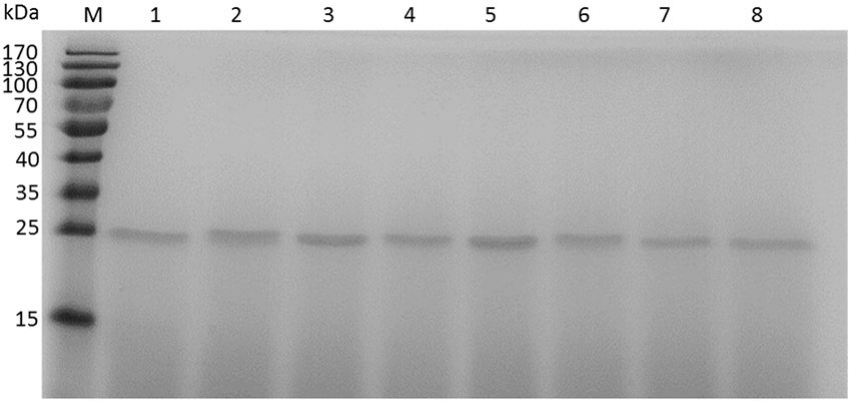
SDS-PAGE analysis of the purified xylanases. M, protein standard; lanes 1–8, wild-type xylanase, T53C-T142C, T46P, S136P, T53C-T142C/T46P, T53C-T142C/S136P, T46P-S136P and T53C-T142C/T46P/S136P.

Wild-type xylanase (xynA-wt) and all mutants (T46P, S136P, T46P/S136P, T53C-T142C, T53C-T142C/T46P, T53C-T142C/S136P and T53C-T142C/T46P/S136P) were expressed in *P. Pastoris* and purified by chromatography, as Fig. 2 shows for the following analysis.

The copy numbers of xynA-wt and mutant T53C-T142C/T46P are the same in recombined *P. Pastoris* genome using relative quantification real-time PCR determination (Table 2).

**Table 2:** The copy number of xynA-wt and T53C-T142C/T46P in *P. Pastoris* genome.

Strain	XynA C_T_^a^	GAP C_T_^a^	ΔC_T_(Avg.XynA CT-Avg.GAP C_T_)^a^	ΔΔC_T_(ΔC_T_-ΔC_T T53C-T142C/T46P_)^a^	Normalized amount
T53C-T142C/T46P	14.77 ± 0.15	14.70 ± 0.14	0.07 ± 0.06	0.00 ± 0.06	1.00 (0.96–1.04)
xynA-wt	12.90 ± 0.08	12.86 ± 0.15	0.03 ± 0.10	−0.03 ± 0.10	1.02 (0.91–1.05)

^a^Values are means ± standard deviation. (n = 3).

### Biochemical characterization of the mutant enzymes

**Figure 3:**
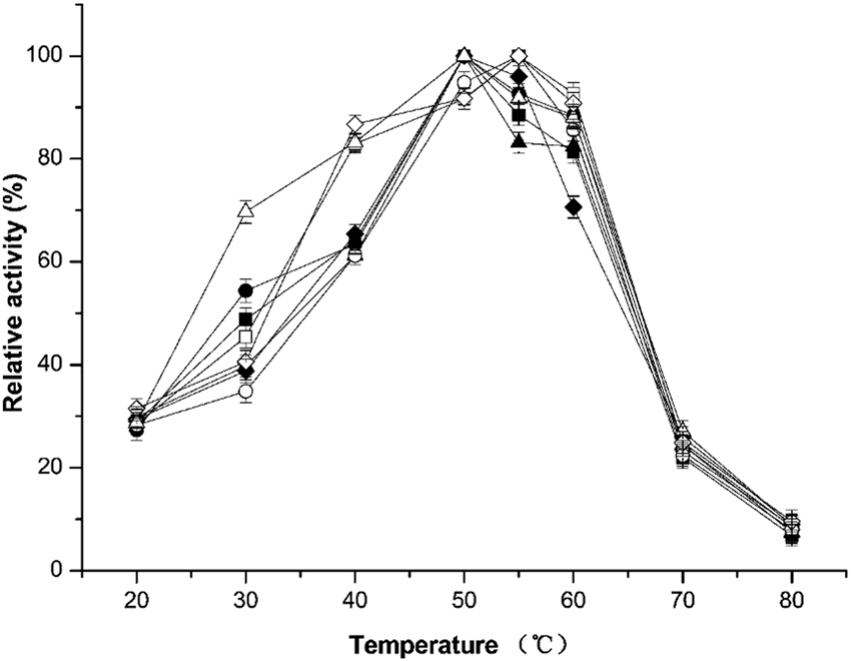
Effect of temperature on the activity of wild-type xylanase and its mutants. The purified xylanases were treated at temperatures ranging from 20 °C to 80 °C with Na_2_HPO_4_-citric acid buffer for 20 min and then assayed at optimum conditions. ■, wild-type xylanase; ●, T53C-T142C; ▲, T46P; ♦, S136P; □, T53C-T142C/T46P; ○, T53C-T142C/S136P; ∆, T46P-S136P; ◊, T53C-T142C/T46P/S136P.

The temperature effect on xylanase activity was demonstrated in Fig. 3. All the double mutants’ optimum temperature was 55 °C except for the mutant T46P/S136P, which kept the same optimum temperature as the single mutants and wild-type xylanase at 50 °C. In the range of 40 °C–60 °C, all the mutants maintained more than 60% of their maximal activity and showed higher relative activity than wild-type xylanase at 60 °C, 70 °C and 80 °C.

We also assayed the thermostability of these mutants as represented by their half-lives (t_1/2_) at 60 °C, 70 °C and 80 °C (Table 3). The results showed that, at 60 °C, all the mutants had improved thermostability compared to the wild-type xylanase. The Mutants T53C-T142C and T53C-T142C/T46P improved the most. Their half-lives at 60 °C were increased up to 18-fold and 22-fold, respectively. At 70 °C and 80 °C, only these two mutants kept residual activity. The half-life of Mutant T53C-T142C was 8 min at 70 °C and 0.5 min at 80 °C, while Mutant T53C-T142C/T46P’s half-life was 9.5 min at 70 °C and 1.6 min at 80 °C, which further increased the thermostability.

**Table 3:** Half-life of *A. sulphureus* acidic xylanase and its mutants in thermal inactivation.

Enzyme	*t*_1/2_ (min)		
	60 °C	70 °C	80 °C
xynA-wt	1.8	ND^a^	ND^a^
T46P	2.5	0.3	ND^a^
S136P	2.1	0.2	ND^a^
T46P/S136P	1.9	ND^a^	ND^a^
T53C-T142C	32.5	8.0	0.5
T53C-T142C/T46P	39.6	9.5	1.6
T53C-T142C/S136P	8.0	1.0	ND^a^
T53C-T142C/T46P/S136P	7.4	0.5	ND^a^

^a^Not detectable.

**Figure 4:**
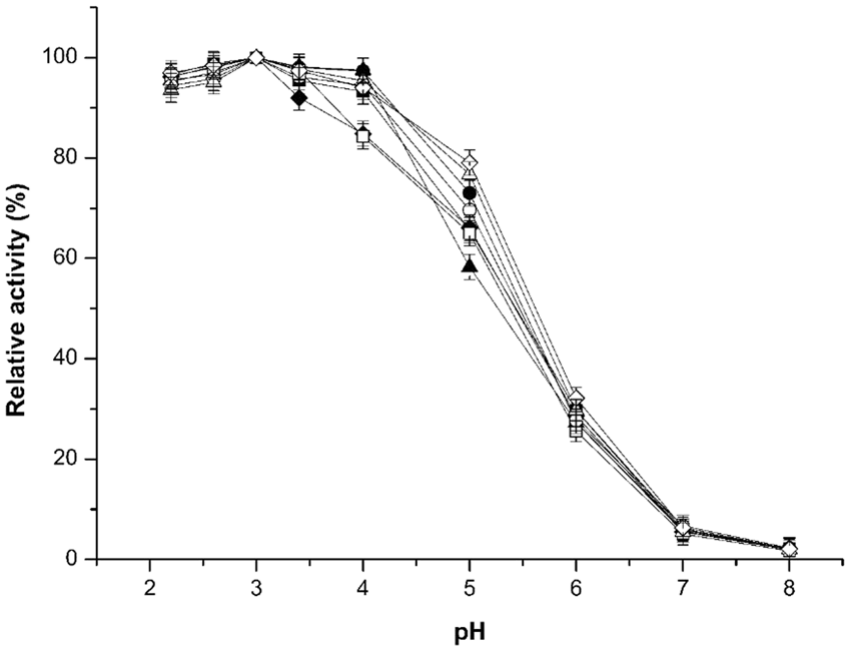
Optimum pH of the wild-type xylanase and its mutants. The purified xylanases were treated under the following conditions and then assayed under optimum conditions. The purified xylanases were assayed at pH values from 2.2 to 8.0 in Na_2_HPO_4_-citric acid buffer. ■, wild-type xylanase; ●, T53C-T142C; ▲, T46P; ♦, S136P; □, T53C-T142C/T46P; ○, T53C-T142C/S136P; ∆, T46P-S136P; ◊, T53C-T142C/T46P/S136P.

**Figure 5:**
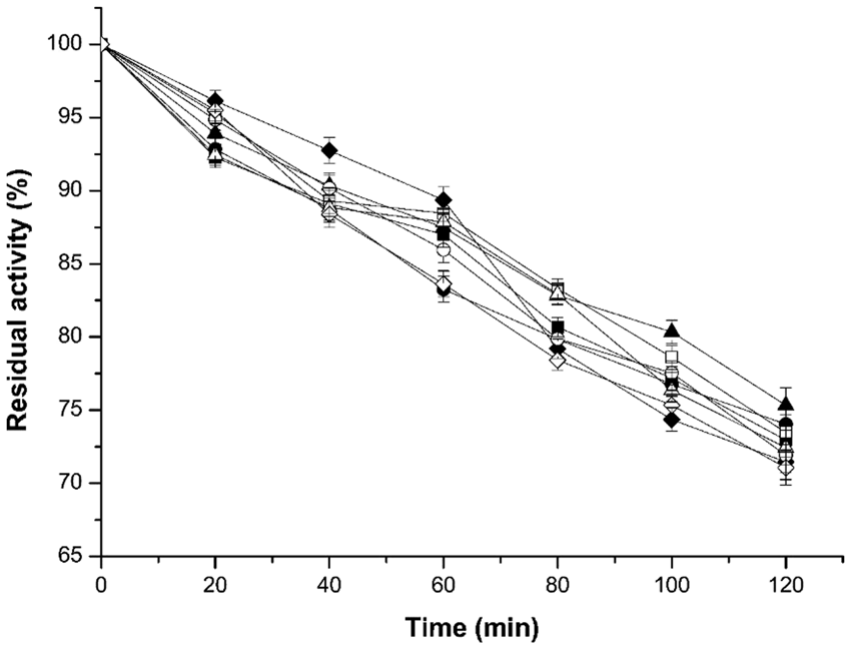
Acidic stability of wild-type xylanase and its mutants. The purified xylanases were diluted in Na_2_HPO_4_-citric acid buffer (pH 2.2) and incubated for 20 min, 40 min, 60 min, 80 min, 100 min and 120 min at room temperature. ■, wild-type xylanase; ●, T53C-T142C; ▲, T46P; ♦, S136P; □, T53C-T142C/T46P; ○, T53C-T142C/S136P; ∆, T46P-S136P; ◊, T53C-T142C/T46P/S136P.

According to the results of optimum pH assays (Fig. 4), we found that for all enzymes, including wild-type xylanase and the mutants, the optimum pH showed no difference at pH 3.0. This demonstrates that the mutations of disulphide bridges and proline substitution had little impact on the enzyme’s optimum pH value. In the range of pH 2.2 to 3.4, all enzymes showed high catalytic activity. However, when the pH value exceeded 4.0, the activity of these enzymes significantly declined. Under weak basic conditions (pH 8.0), the enzymes showed very low activity. Simultaneously, the wild type xylanase and all mutants displayed hyper-resistance to an acidic environment. After incubation at pH 2.2 for 120 min, their residual activity remained at over 70% of the full activity (Fig. 5).

Effects of various metal ions and chemical reagents on the activity of xylanases were examined and summarized in Table 4. Most of the xylanases were prominently suppressed by Ca^2+^ and Cu^2+^. The ions Zn^2+^ and Mn^2+^ were observed to stimulate the activity of the xylanases (approximately 1.1–1.5-fold of the original activity), while the ions Na^+^, K^+^, Mg^2+^ and EDTA inhibited the enzymes by 5–20% of their activity.

**Table 4:** Effects of metal ions and chemical reagents (10 mM) on β-xylanase activity.

Relative activity (%)	control	NaCl^a^	KCl^a^	CaCl_2_^a^	MgCl_2_^a^	CuSO_4_^a^	ZnSO_4_^a^	MnSO_4_^a^	EDTA^a^
xynA-wt	100	89.0 ± 0.4	89.0 ± 0.3	56.9 ± 0.5	95.1 ± 0.5	20.9 ± 0.6	129.8 ± 0.5	146.5 ± 0.6	85.5 ± 0.5
T53C-T142C	100	92.3 ± 0.6	92.3 ± 0.4	59.6 ± 0.4	81.7 ± 0.3	29.6 ± 0.5	109.8 ± 0.5	144.3 ± 0.4	91.0 ± 0.6
T46P	100	66.5 ± 0.5	83.6 ± 0.5	98.6 ± 0.3	90.6 ± 0.7	16.2 ± 0.4	111.7 ± 0.5	131.4 ± 0.3	89.2 ± 0.5
S136P	100	96.5 ± 0.3	87.4 ± 0.3	79.1 ± 0.4	86.1 ± 0.3	16.1 ± 0.6	137.7 ± 0.5	147.7 ± 0.4	91.8 ± 0.4
T53C-T142C/T46P	100	84.6 ± 0.4	93.1 ± 0.4	63.0 ± 0.3	89.2 ± 0.5	35.2 ± 0.5	139.5 ± 0.6	137.4 ± 0.4	91.6 ± 0.5
T53C-T142C/S136P	100	90.1 ± 0.6	82.2 ± 0.4	48.6 ± 0.6	88.0 ± 0.5	13.7 ± 0.4	112.7 ± 0.6	110.2 ± 0.5	83.9 ± 0.3
T46P/S136P	100	95.9 ± 0.7	81.9 ± 0.3	78.9 ± 0.3	91.4 ± 0.6	58.1 ± 0.4	120.8 ± 0.5	131.4 ± 0.7	93.4 ± 0.4
T53C-T142C/T46P/S136P	100	89.5 ± 0.5	93.2 ± 0.4	80.5 ± 0.5	95.1 ± 0.5	80.4 ± 0.3	137.9 ± 0.6	133.4 ± 0.5	96.0 ± 0.5

^a^Values are means ± standard deviation.

The specific activity and kinetic values of the xylanases were determined with beech wood xylan as the substrate (Table 5). Compared with the specific activity of wild-type xylanase (237.7 U/mg), all mutants showed decreased specific activity, ranging from 138.4 to 229.4 U/mg. Moreover, the introduced disulphide bridges and proline residue substitutions had impacts on the kinetics of all mutants. Except for T53C-T142C, all other mutants were found to have obviously decreased *K*_*m*_ and *V*_max_ values. The results indicated that both the introduced disulphide bridge (T53C-T142C) and the proline residue substitutions (T46P and S136P) decreased the catalytic efficiency of these mutant enzymes.

**Table 5:** Specific activity and kinetics of wild-type xylanase and its mutants.

Enzyme	Sp act (U/mg)^a^	*K*_*m*_ (mg/mL)^a^	*V*_max_ (μmol/min/mg)^a^
xynA-wt	237.7 ± 2.1	2.3 ± 0.5	454.5 ± 12.1
T46P	220.5 ± 1.9	1.0 ± 0.2	344.8 ± 9.6
S136P	162.2 ± 2.6	1.2 ± 0.4	263.2 ± 8.7
T46P/S136P	138.4 ± 3.1	0.7 ± 0.04	196.1 ± 6.4
T53C-T142C	229.4 ± 3.4	3.6 ± 0.9	434.8 ± 11.8
T53C-T142C/T46P	217.9 ± 2.3	1.2 ± 0.3	303.0 ± 8.5
T53C-T142C/S136P	188.9 ± 1.7	1.5 ± 0.6	263.2 ± 5.6
T53C-T142C/T46P/S136P	146.0 ± 1.8	1.8 ± 0.7	243.9 ± 7.3

^a^Values are means ± standard deviation. Sp act: Specific activity.

### Fermentation in a 10-L Bioreactor

The recombinant yeast expressing T53C-T142C/T46P was cultured in a 10-L bioreactor. The maximal xylanase activity reached 1,684 U/mL, with an enzyme protein concentration of 7.73 mg/mL after methanol induction for 96 h. Comparatively, the fermentation activity of the wild-type xylanase reached 3,900 U/mL with 16.41 mg/mL of enzyme protein concentration under the same induction conditions. Thus, room to improve the fermentation activity by optimizing the fermentation conditions still exists.

## Discussion

Mutagenesis studies have shown that the properties of disulphide bridges of proteins have a major role in the thermostability of enzymes^15, 16^. Disulphide bridges are believed to stabilize proteins mostly through an entropic effect by decreasing the entropy of the protein’s unfolded state^17^. Le *et al*. improved the thermostability of CalB (Lipase B from *Candida antarctica*) through the introduction of a new disulphide bridge A162C-K308C. The half-life of CalB A162C-K308C was 4.5-fold higher than that of its wild-type counterpart^18^. Yu *et al*. introduced a disulphide bridge between F95C and F214C into the lipase from *Rhizopus chinensis* in the hinge region of the lid. The disulphide variant improved the thermostability with an 11-fold increase in the t_1/2s_ value at 60 °C and 70 °C^19^.

In the present study, we selected three pairs of amino acids to mutate into cysteine in order to introduce three pairs of disulphide bridges. Only Mutant T53C-T142C significantly enhanced heat resistance. The presence of the disulphide bridge 53–142 was confirmed by quickly measuring protein sulphydryls. As Fig. 1 demonstrates, the disulphide bridge T53C-T142C is situated at the β-sheet of the xylanase tertiary structure, while the disulphide bridges P51C-T144C and S49C-A146C are located in the random coil of the xylanase protein, which is structurally less stable than β-sheet and liable to configure through the interaction of side chains^20^. This is possibly the reason the disulphide bridges P51C-T144C and S49C-A146C were unsuccessfully constructed to increase the enzyme’s thermostability. The half-life of Mutant T53C-T142C at 60 °C increased up to 18-fold, which was 8 min and 0.5 min at 70 °C and 80 °C compared to that of wild-type xylanase.

Proteins can also be stabilized by decreasing their entropy of unfolding^21^. Prolines, with their pyrrolidine ring, can only adopt a few configurations to decrease a protein’s entropy^22^. Additionally, proline can bend the polypeptide on itself to make it much easier for the backbone to form hydrogen bonds with polar side chains of other turns and loops^23^, which contribute to the improvement of turn stabilization. In this study, we introduced prolines at 9 positions (D32P, G33P, S35P, T45P, T46P, Y75P, S136P, S160P and D161P), which were distributed at several random coils (Fig. 1). Although the mutations failed to change the optimum catalytic conditions of the original enzyme, the improvement of thermostability appeared less significant than our expectation. Except for Mutants T46P and S136P, the other 7 mutant enzymes showed no improvement in thermostability. However, in documented studies^24–26^, introduction of proline residues at certain positions has been reported to be effective at improving the thermostability of many enzymes, in which thermal stability was enhanced 10 °C on average. Due to many factors that affect protein thermostability, substituted prolines cannot enhance heat resistance markedly all the time^27^.

To determine whether introduction of both disulphide bridges and proline residues can enhance the thermostability of xylanase, we designed double mutations. The results of thermal stability assays revealed that the combination of two proline residues (T46P/S136P and T53C-T142C/T46P/S136P) failed to increase the thermostability compared to its single-mutation counterparts. On the other hand, mutant T53C-T142C/T46P significantly improved the heat resistance better than T53C-T142C/S136P, even better than T53C-T142C. The gain of extra thermostability is apparently contributed by the mutation of T46P, which is located on the edge of β-sheets and might increase the integrity of the local structure. Moreover, the optimal temperatures of T53C-T142C/T46P, T53C-T142C/S136P and T53C-T142C/T46P/S136P were increase 5 °C than the wild type enzyme and the single mutants. It’s also worth mentioning that the resistance to Ca^2+^ and Cu^2+^ of T46P/S136P and T53C-T142C/T46P/S136P were much more remarkable than other mutants.

Improved xylanases with single and double mutations showed significant improvement in enzyme thermostability. These characteristics make them favourable for wide industrial application, especially in the process of feed pelleting. However, introduced disulphide bridges (T53C-T142C) and proline residue substitutions (T46P and S136P) both decreased the catalytic efficiency of these mutant enzymes. It demonstrated that it was necessary to note the balance between activity and stability, and the increased stability of the mutants will incur the expense of catalytic activity^28^. The productivity and stability also need to be balanced. In this study, the productivity of mutant T53C-T142C/T46P decreased comparing with xynA-wt even though their fragment copy numbers were the same in the recombined *P. Pastoris* genome. The disulphide bond introduction might cause the mutant incorrect folding intracellularly in *P. Pastoris*, especially at high growth rate^29^. Refolding or degradation of misfolded mutant protein could slow down the protein synthesis and process further. Therefore, the optimal fermentation parameters of xynA-wt could not seems to be suitable for mutant T53C-T142C/T46P. Controlling recombined *P. Pastoris* of mutant T53C-T142C/T46P at low growth rate to avoid misfolded protein should help increasing its productivity.

Thermostability is essential to keep enzyme activity after feed pelleting. The engineered xylanase T53C-T142C/T46P, with the combined introduction of the disulphide bond and proline substitution, improved the enzyme’s thermostability. It can be a prospective additive in feed manufacture.

## Materials and Methods

### Strains, vectors, media and chemicals

*Escherichia coli* Top 10 (Tiangen, China) and *P. pastoris* X-33 (Invitrogen, USA) were used as the host strains. pPICZαA-xynA-opt containing the optimized *A. sulphureus* xylanase gene xynA-opt (GenBank accession number: EF114744) was stored by our lab. Xylan and DTNB (5,5′-Dithiobis (2-nitrobenzoic acid)) were obtained from Sigma (USA). All other chemicals not specifically mentioned were of analytical grade and commercially available.

### Mutagenesis of xynA

A xylanase from *Aspergillus niger* (PDB 2QZ2_A) carrying 98% amino-acid identity with *A. sulphureus* xylanase was used as a structure model to predict the mutant sites. According to the prediction by Coot^14^, three pairs of disulphide bond mutants (S49C and A146C, P51C and T144C, T53C and T142C) and nine proline substitutions (D32P, G33P, S35P, T45P, T46P, Y75P, S136P, S160P, D161P) were designed. XynA-wt and designed mutants’ DNA sequences were displayed in Supplementary Information as well as the translated amino acids sequences. The gene mutations were generated by over-lapped PCR with designed primers (Table 6) and transformed into *E. coli* Top 10. The recombinant plasmids were confirmed by DNA sequencing.

**Table 6:** Oligonucleotide primers used in this study.

Name	Sequence
xynA-F	5′-CGGAATTCTCCGCTGGTATCAAC-3′
xynA-R	5′-GCTCTAGATTAGGAGGAGATAG-3′
49-146-F	5′-TCCTGTAACCCAATCACTTAC-3′
49-146-R	5′-GGTTACAAACAGTAACAGTACC-3′
51-144-F	5′-CCAACTGTATCACTTACTCCG-3′
51-144-R	5′-AGCAACACAAACAGTACCGGAAG-3
53-142-F	5′-CTGTTACTCCGCTGACTACTC-3′
53-142-R	5′-AACACAACCGGAAGTTCTAGTG-3′
D32p-F	5′-GGGAGCCAGGTGTTTCCTCC-3′
D32p-R	5′-ACACCTGGCTCCCAGTACAT-3′
G33p-F	5′-AGGACCCTGTTTCCTCCGAC-3′
G33p-R	5′-GAAACAGGGTCCTCCCAGTA-3′
S35p-F	5′-GTGTTCCTTCCGACTTCGTT-3′
S35p-R	5′-TCGGAAGGAACACCGTCCTC-3′
T45p-F	5′-GTTGGCCTACTGGTTCCTCC-3′
T45p-R	5′-CCAGTAGGCCAACCCAAACC-3′
T46p-F	5′- GGACTCCTGGTTCCTCCAAC-3′
T46p-R	5′-GAACCAGGAGTCCAACCCAA-3′
Y75p-F	5′-TTAACCCACCACAAGCTGAG-3′
Y75p-R	5′-TGTGGTGGGTTAACCCAACC-3′
S136p-F	5′-GAGAGCCTACTAGAACTTCC-3′
S136p-R	5′-CTAGTAGGCTCTCTAACGGA-3′
S160p-F	5′-GTAACCCTGACTTCAACTAC-3′
S160p-R	5′-AAGTCAGGGTTACCGAAACC-3′
D161p-F	5′-ACTCCCCATTCAACTACCAA-3′
D161p-R	5′-TTGAATGGGGAGTTACCGAA-3′
GAP ref F	5′-AGTCCACCGGTGTTTTCACC-3′
GAP ref R	5′-GTTGACAACCTTGGCCAATG-3′
xynA qPCR F	5′-GGTTCCTCCTCCTACTTGGC-3′
xynA qPCR R	5′-GGAGAAGTATTGAGTGAAAGTGGAAG-3′

### Transformation, shake-flask expression and purification of xylanase

The recombinant plasmids were linearized with endonuclease *Sac* I and transformed into *P. pastoris* X-33 cells by a Gene Pulser Xcell™ Electroporation System (Bio-Rad, Hercules, CA, USA) working at 2,000 V and 5 ms. The transformants were screened on YPDS plates (10 g/L yeast extract, 20 g/L peptone, 20 g/L dextrose, 1 mol/L sorbitol, and 20 g/L agar) containing 100 μg/mL of Zeocin for 2–3 days.

Recombinant yeast culture and protein expression were conducted according to the method of Cao *et al*.^13^. The supernatant of the culture was collected by centrifugation at 5,000 rpm for 5 min, followed by precipitation with ammonium sulphate of 75% saturation degree on ice. The precipitate was collected by centrifugation at 12,000 rpm for 10 min and dissolved in 6 mL of 50 mM HAc-NaAc buffer (pH 5.3), then dialysed in 50 mM HAc-NaAc buffer (pH 5.3) in order to remove ammonium sulphate. After dialysis, the crude enzyme was purified via strong anion exchange column (UNOsphere Q, Bio-Rad) by gradient elution with 0–1 M NaCl (ÄKTA™ pure system, GE Healthcare). The active fractions were pooled and stored at 4 °C for further analysis. Protein concentration was determined with a Pierce^TM^ BCA Protein Assay Kit (Thermo, USA).

### Assay of xylanase activity

The substrate was 0.8% (w/v) of xylan (from beechwood, Sigma X4252). The xylanase activity was assayed according to the method of Lu *et al*.^30^. The liberated reducing sugar was measured by the dinitrosalicylic acid (DNS) method^31^. Each experiment was performed in triplicate. One unit of xylanase activity was defined as the amount of the enzyme that released 1 μmol of xylose per minute under the assay conditions.

### Fermentation in 10-L Bioreactor

The recombinant *P. pastoris* expressing T53C-T142C/T46P was cultured in a 10-L Bioreactor according to the method of Lv *et al*.^32^. Briefly, The yeast was cultured in basal salt medium (containing 50 g glucose, 5 g KH_2_PO_4_, 0.93 g CaSO_4_, 18.2 g K_2_SO_4_, 14.9 g MgSO_4_, 1.5 g KOH, and 50 g NH_4_H_2_PO_4_/L) in a 10-L bioreactor for 20–24 hr, 50% (w/v) glycerol was added into the medium after the glucose was exhausted, and the carbon source was switched to methanol when the biomass amounted to over 200 mg/mL to induce xylanase expression.

### Biochemical characterization of xylanase

The purified enzyme was diluted in Na_2_HPO_4_-citric acid buffer (pH 3.4). The determination of optimum temperature was conducted in the temperature range from 20 °C to 80 °C at intervals of 10 °C. The maximal activity was defined as 100% of relative enzyme activity.

The thermostability was determined by measuring the half-life (t_1/2_) of the enzyme, which is defined as the time that the enzyme activity declines to half of the full activity at a certain temperature. We adopted three temperatures (60 °C, 70 °C and 80 °C) with other optimum conditions.

For determining optimum pH for enzyme activity, the enzyme was diluted with Na_2_HPO_4_-citric acid buffers with pH range from 2.0 to 8.0. The xylanase activity was assayed at 50 °C.

For the determination of enzyme stability in acidic condition, the recombinant xylanase was diluted with Na_2_HPO_4_-citric acid buffer at pH 2.2 and incubated for 20 min, 40 min, 60 min, 80 min, 100 min and 120 min at 4 °C, followed by further dilution with Na_2_HPO_4_-citric acid buffer at the optimum pH value. The residual activity was measured at standard condition.

Effects of various metal ions and chemical reagents on the activity of the enzyme were tested with a final concentration of ions of 10 mM (CaCl_2_, CuSO_4_, MnSO_4_, MgCl_2_, ZnSO_4_, NaCl, KCl, EDTA). Xylanase was pre-incubated with each type of metal ion or chemical for 2 h, after which the residual activity was determined at the optimum conditions for 20 min. The reaction mixture without metal ions or chemical reagents was used as the control.

The kinetic parameters were assayed by incubation of the enzyme with a series of concentrations of the substrate xylan (1 mg/mL to 8 mg/mL at the interval of 1 mg/mL) in Na_2_HPO_4_-citric acid buffer at the optimum conditions.

### Quick measurement of protein sulphydryls

The analysis of protein sulphydryls was performed according to the method of Riener *et al*.^33^. Briefly, the gradient concentrations of cysteine solutions prepared by dilution of standard cysteine using 0.25 mol/L of Tris-HCl were 0, 0.025, 0.05, 0.1, 0.15 and 0.2 mmol/L. One mL of each diluted cysteine solution was thoroughly pre-warmed in a water bath at 25 °C for 10 min followed by an assay of OD values at the wave length of 412 nm. Subsequently, the enzyme solution and DTNB were added to each cysteine solution. The mixtures were temperature balanced in a water bath at 25 °C for 10 min, and then the ODs were assayed at 412 nm. According to the curve of the cysteine standard, the concentrations of free mercapto group were calculated.

### Copy number of xynA-wt and T53C-T142C/T46P in *P. Pastoris* genome

Relative quantification real-time PCR was performed to determinate the copy number of xynA-wt and T53C-T142C/T46P in the genome of recombined *P. Pastoris*. Two pairs of primers were designed to amplify the glyceraldehyde-3-phosphate dehydrogenase (GAP) gene as reference gene, and to amplify the xynA-wt or the T53C-T142C/T46P gene, namely GAP ref F/R and xynA qPCR F/R respectively. The relative quantification real-time PCR mixture of 10.0 μL was prepared using the SYBR^®^ Fast qPCR Mix (TAKARA BIO INC): 5.0 μL SYBR Fast qPCR Mix (2×), 0.2 μL of each primer (10 μM), 1.0 μL template DNA (50 ng), and PCR-grade water up to 10.0 μL. The thermal cycling was set as follows: 5 min initial denaturation at 95 °C, 35 cycles of 30 s denaturation at 95 °C, 30 s annealing at 54.5 °C and 30 s extension at 72 °C. The fluorescence signal was detected at the end of each extension step to determine the threshold cycle (C_T_). The copy number of xynA-wt and T53C-T142C/T46P in the recombined *P. Pastoris* genome were calculated by ΔΔC_T_ method^34^.

## Electronic supplementary material

DNA and translated amino acids sequences of xynA-wt and designed mutants
