# Supplementary material for: Improved thermostability of an acidic xylanase from Aspergillus sulphureus by combined disulphide bridge introduction and proline residue substitution
Source: Sci Rep. 2017 May 8;7:1587. doi: 10.1038/s41598-017-01758-5 (PMC5431495; doi:10.1038/s41598-017-01758-5)
Supplement: Supplementary file 1 — DNA and translated amino acids sequences of xynA-wt and designed mutants [file 41598_2017_1758_MOESM1_ESM.pdf]

Supplementary Information for

Improved thermostability of an acidic xylanase from *Aspergillus sulphureus* by combined disulphide bridge introduction and proline residue substitution

Wenhan Yang[1], Yongzhi Yang[1,†] , Lingdi Zhang[2], Hang Xu[3], Xiaojing Guo[1], Xu Yang[1], Bing Dong[1] & Yunhe Cao[1,*]

1, State Key Laboratory of Animal Nutrition, China Agricultural University, No.2 Yuanmingyuan West Road, Beijing 100193, China.

2, School of Medicine Biochemistry and Molecular Genetics, University of Colorado Denver, Aurora, CO 80045.

3, Institute of Biophysics, Chinese Academy of Sciences, Beijing 100101, China.

† The first two authors contributed equally to this paper

* Correspondence and requests for materials should be addressed to Y.H.C. (email: caoyh@cau.edu.cn)

This file includes:

- DNA sequences of xynA-wt and designed mutants
- Translated amino acids sequences of xynA-wt and designed mutants

DNA sequences of xynA-wt and designed mutants

```
xynA-opt                  TCCGCTGGTATCAACTACGTTCAAAACTACAACGGTAACTTGGGTGACTT    50
D32P                      --------------------------------------------------    50
G33P                      --------------------------------------------------    50
S35P                      --------------------------------------------------    50
T45P                      --------------------------------------------------    50
T46P                      --------------------------------------------------    50
Y75P                      --------------------------------------------------    50
S136P                     --------------------------------------------------    50
S160P                     --------------------------------------------------    50
D161P                     --------------------------------------------------    50
S49C-A146C                --------------------------------------------------    50
P51C-T144C                --------------------------------------------------    50
T53C-T142C                --------------------------------------------------    50
T53C-T142C/T46P           --------------------------------------------------    50
T53C-T142C/S136P          --------------------------------------------------    50
T53C-T142C/T46P/S136P     --------------------------------------------------    50
T46P/S136P                --------------------------------------------------    50

xynA-opt                  CACTTACGACGAGTCCGCTGGTACTTTCTCCATGTACTGGGAGGACGGTG    100
D32P                      -------------------------------------------cca----    100
G33P                      ----------------------------------------------cc--    100
S35P                      --------------------------------------------------    100
T45P                      --------------------------------------------------    100
T46P                      --------------------------------------------------    100
Y75P                      --------------------------------------------------    100
S136P                     --------------------------------------------------    100
S160P                     --------------------------------------------------    100
D161P                     --------------------------------------------------    100
S49C-A146C                --------------------------------------------------    100
P51C-T144C                --------------------------------------------------    100
T53C-T142C                --------------------------------------------------    100
T53C-T142C/T46P           --------------------------------------------------    100
T53C-T142C/S136P          --------------------------------------------------    100
T53C-T142C/T46P/S136P     --------------------------------------------------    100
T46P/S136P                --------------------------------------------------    100
```

```
xynA-opt                  TTTCCTCCGACTTCGTTGTTGGTTTGGGTTGGACTACTGGTTCCTCCAAC   150
D32P                      --------------------------------------------------   150
G33P                      --------------------------------------------------   150
S35P                      --c-t---------------------------------------------   150
T45P                      --------------------------------c-----------------   150
T46P                      -----------------------------------c--------------   150
Y75P                      --------------------------------------------------   150
S136P                     --------------------------------------------------   150
S160P                     --------------------------------------------------   150
D161P                     --------------------------------------------------   150
S49C-A146C                ---------------------------------------------gt---   150
P51C-T144C                --------------------------------------------------   150
T53C-T142C                --------------------------------------------------   150
T53C-T142C/T46P           -----------------------------------c--------------   150
T53C-T142C/S136P          --------------------------------------------------   150
T53C-T142C/T46P/S136P     -----------------------------------c--------------   150
T46P/S136P                -----------------------------------c--------------   150

xynA-opt                  CCAATCACTTACTCCGCTGACTACTCCGCTTCCGGTTCCTCCTCCTACTT   200
D32P                      --------------------------------------------------   200
G33P                      --------------------------------------------------   200
S35P                      --------------------------------------------------   200
T45P                      --------------------------------------------------   200
T46P                      --------------------------------------------------   200
Y75P                      --------------------------------------------------   200
S136P                     --------------------------------------------------   200
S160P                     --------------------------------------------------   200
D161P                     --------------------------------------------------   200
S49C-A146C                --------------------------------------------------   200
P51C-T144C                tgt-----------------------------------------------   200
T53C-T142C                ------tg------------------------------------------   200
T53C-T142C/T46P           ------tg------------------------------------------   200
T53C-T142C/S136P          ------tg------------------------------------------   200
T53C-T142C/T46P/S136P     ------tg------------------------------------------   200
T46P/S136P                --------------------------------------------------   200
```

```
xynA-opt               GGCTGTTTACGGTTGGGTTAACTACCCACAAGCTGAGTACTACATCGTTG   250
D32P                   --------------------------------------------------   250
G33P                   --------------------------------------------------   250
S35P                   --------------------------------------------------   250
T45P                   --------------------------------------------------   250
T46P                   --------------------------------------------------   250
Y75P                   ----------------------cca-------------------------   250
S136P                  --------------------------------------------------   250
S160P                  --------------------------------------------------   250
D161P                  --------------------------------------------------   250
S49C-A146C             --------------------------------------------------   250
P51C-T144C             --------------------------------------------------   250
T53C-T142C             --------------------------------------------------   250
T53C-T142C/T46P        --------------------------------------------------   250
T53C-T142C/S136P       --------------------------------------------------   250
T53C-T142C/T46P/S136P  --------------------------------------------------   250
T46P/S136P             --------------------------------------------------   250

xynA-opt               AGGACTACGGTGACTACAACCCATGTTCCTCCGCTACTTCCTTGGGTACT   300
D32P                   --------------------------------------------------   300
G33P                   --------------------------------------------------   300
S35P                   --------------------------------------------------   300
T45P                   --------------------------------------------------   300
T46P                   --------------------------------------------------   300
Y75P                   --------------------------------------------------   300
S136P                  --------------------------------------------------   300
S160P                  --------------------------------------------------   300
D161P                  --------------------------------------------------   300
S49C-A146C             --------------------------------------------------   300
P51C-T144C             --------------------------------------------------   300
T53C-T142C             --------------------------------------------------   300
T53C-T142C/T46P        --------------------------------------------------   300
T53C-T142C/S136P       --------------------------------------------------   300
T53C-T142C/T46P/S136P  --------------------------------------------------   300
T46P/S136P             --------------------------------------------------   300
```

```
xynA-opt               GTTTACTCCGACGGTTCCACTTACCAAGTTTGTACTGACACTAGAACTAA   350
D32P                   --------------------------------------------------   350
G33P                   --------------------------------------------------   350
S35P                   --------------------------------------------------   350
T45P                   --------------------------------------------------   350
T46P                   --------------------------------------------------   350
Y75P                   --------------------------------------------------   350
S136P                  --------------------------------------------------   350
S160P                  --------------------------------------------------   350
D161P                  --------------------------------------------------   350
S49C-A146C             --------------------------------------------------   350
P51C-T144C             --------------------------------------------------   350
T53C-T142C             --------------------------------------------------   350
T53C-T142C/T46P        --------------------------------------------------   350
T53C-T142C/S136P       --------------------------------------------------   350
T53C-T142C/T46P/S136P  --------------------------------------------------   350
T46P/S136P             --------------------------------------------------   350

xynA-opt               CGAGCCATCCATCACTGGTACTTCCACTTTCACTCAATACTTCTCCGTTA   400
D32P                   --------------------------------------------------   400
G33P                   --------------------------------------------------   400
S35P                   --------------------------------------------------   400
T45P                   --------------------------------------------------   400
T46P                   --------------------------------------------------   400
Y75P                   --------------------------------------------------   400
S136P                  --------------------------------------------------   400
S160P                  --------------------------------------------------   400
D161P                  --------------------------------------------------   400
S49C-A146C             --------------------------------------------------   400
P51C-T144C             --------------------------------------------------   400
T53C-T142C             --------------------------------------------------   400
T53C-T142C/T46P        --------------------------------------------------   400
T53C-T142C/S136P       --------------------------------------------------   400
T53C-T142C/T46P/S136P  --------------------------------------------------   400
T46P/S136P             --------------------------------------------------   400
```

```
xynA-opt                 GAGAGTCCACTAGAACTTCCGGTACTGTTACTGTTGCTAACCACTTCAAC   450
D32P                     --------------------------------------------------   450
G33P                     --------------------------------------------------   450
S35P                     --------------------------------------------------   450
T45P                     --------------------------------------------------   450
T46P                     --------------------------------------------------   450
Y75P                     --------------------------------------------------   450
S136P                    -----c-t------------------------------------------   450
S160P                    --------------------------------------------------   450
D161P                    --------------------------------------------------   450
S49C-A146C               -----------------------------------tg-------------   450
P51C-T144C               -----------------------------tg-------------------   450
T53C-T142C               -----------------------tg-------------------------   450
T53C-T142C/T46P          -----------------------tg-------------------------   450
T53C-T142C/S136P         -----c-t---------------tg-------------------------   450
T53C-T142C/T46P/S136P    -----c-t---------------tg-------------------------   450
T46P/S136P               -----c-t------------------------------------------   450

xynA-opt                 TTCTGGGCTCAACACGGTTTCGGTAACTCCGACTTCAACTACCAAGTTGT   500
D32P                     --------------------------------------------------   500
G33P                     --------------------------------------------------   500
S35P                     --------------------------------------------------   500
T45P                     --------------------------------------------------   500
T46P                     --------------------------------------------------   500
Y75P                     --------------------------------------------------   500
S136P                    --------------------------------------------------   500
S160P                    ---------------------------c-t--------------------   500
D161P                    ------------------------------cca-----------------   500
S49C-A146C               --------------------------------------------------   500
P51C-T144C               --------------------------------------------------   500
T53C-T142C               --------------------------------------------------   500
T53C-T142C/T46P          --------------------------------------------------   500
T53C-T142C/S136P         --------------------------------------------------   500
T53C-T142C/T46P/S136P    --------------------------------------------------   500
T46P/S136P               --------------------------------------------------   500
```

```
xynA-opt                TGCTGTTGAGGCTTGGTCCGGTGCTGGTTCCGCTTCCGTTACTATCTCCT   550
D32P                    --------------------------------------------------   550
G33P                    --------------------------------------------------   550
S35P                    --------------------------------------------------   550
T45P                    --------------------------------------------------   550
T46P                    --------------------------------------------------   550
Y75P                    --------------------------------------------------   550
S136P                   --------------------------------------------------   550
S160P                   --------------------------------------------------   550
D161P                   --------------------------------------------------   550
S49C-A146C              --------------------------------------------------   550
P51C-T144C              --------------------------------------------------   550
T53C-T142C              --------------------------------------------------   550
T53C-T142C/T46P         --------------------------------------------------   550
T53C-T142C/S136P        --------------------------------------------------   550
T53C-T142C/T46P/S136P   --------------------------------------------------   550
T46P/S136P              --------------------------------------------------   550

xynA-opt                CCTAA   555
D32P                    -----   555
G33P                    -----   555
S35P                    -----   555
T45P                    -----   555
T46P                    -----   555
Y75P                    -----   555
S136P                   -----   555
S160P                   -----   555
D161P                   -----   555
S49C-A146C              -----   555
P51C-T144C              -----   555
T53C-T142C              -----   555
T53C-T142C/T46P         -----   555
T53C-T142C/S136P        -----   555
T53C-T142C/T46P/S136P   -----   555
T46P/S136P              -----   555
```

Translated amino acids sequences of xynA-wt and designed mutants

```
xynA-opt              SAGINYVQNYNGNLGDFTYDESAGTFSMYWEDGVSSDFVVGLGWTTGSSN    50
D32P                  -------------------------------p------------------    50
G33P                  --------------------------------p-----------------    50
S35P                  ----------------------------------p---------------    50
T45P                  --------------------------------------------p-----    50
T46P                  ---------------------------------------------p----    50
Y75P                  --------------------------------------------------    50
S136P                 --------------------------------------------------    50
S160P                 --------------------------------------------------    50
D161P                 --------------------------------------------------    50
S49C-A146C            ------------------------------------------------c-    50
P51C--T144C           --------------------------------------------------    50
T53C-T142C            --------------------------------------------------    50
T53C-T142C/T46P       ---------------------------------------------p----    50
T53C-T142C/S136P      --------------------------------------------------    50
T53C-T142C/T46P/S136P ---------------------------------------------p----    50
T46P/S136P            ---------------------------------------------p----    50

xynA-opt              PITYSADYSASGSSSYLAVYGWVNYPQAEYYIVEDYGDYNPCSSATSLGT   100
D32P                  --------------------------------------------------   100
G33P                  --------------------------------------------------   100
S35P                  --------------------------------------------------   100
T45P                  --------------------------------------------------   100
T46P                  --------------------------------------------------   100
Y75P                  ------------------------p-------------------------   100
S136P                 --------------------------------------------------   100
S160P                 --------------------------------------------------   100
D161P                 --------------------------------------------------   100
S49C-A146C            --------------------------------------------------   100
P51C--T144C           c-------------------------------------------------   100
T53C-T142C            --c-----------------------------------------------   100
T53C-T142C/T46P       --c-----------------------------------------------   100
T53C-T142C/S136P      --c-----------------------------------------------   100
T53C-T142C/T46P/S136P --c-----------------------------------------------   100
T46P/S136P            --------------------------------------------------   100
```

xynA-opt	VYSDGSTYQVCTDTRTNEPSITGTSTFTQYFSVRESTRTSGTVTVANHFN	150
D32P	--	150
G33P	--	150
S35P	--	150
T45P	--	150
T46P	--	150
Y75P	--	150
S136P	-----------------------------------p--------------	150
S160P	--	150
D161P	--	150
S49C-A146C	---c----	150
P51C--T144C	---c------	150
T53C-T142C	---c--------	150
T53C-T142C/T46P	---c--------	150
T53C-T142C/S136P	-----------------------------------p-----c--------	150
T53C-T142C/T46P/S136P	-----------------------------------p-----c--------	150
T46P/S136P	-----------------------------------p--------------	150

xynA-opt	FWAQHGFGNSDFNYQVVAVEAWSGAGSASVTISS	184
D32P	----------------------------------	184
G33P	----------------------------------	184
S35P	----------------------------------	184
T45P	----------------------------------	184
T46P	----------------------------------	184
Y75P	----------------------------------	184
S136P	----------------------------------	184
S160P	---------p------------------------	184
D161P	----------p-----------------------	184
S49C-A146C	----------------------------------	184
P51C--T144C	----------------------------------	184
T53C-T142C	----------------------------------	184
T53C-T142C/T46P	----------------------------------	184
T53C-T142C/S136P	----------------------------------	184
T53C-T142C/T46P/S136P	----------------------------------	184
T46P/S136P	----------------------------------	184
